# An evaluation of the Monarch chemistry analyser

**DOI:** 10.1155/S1463924689000039

**Published:** 1989

**Authors:** D. J. Berry, C. P. Price

**Affiliations:** Department of Clinical Biochemistry Addenbrooke's Hospital Hills Road Cambridge CB2 2QR UK

## Abstract

The Monarch Chemistry system, a centrifugal analyser incorporating sophisticated robotics for analytical rotor transfer and flexible software for workload scheduling, has been evaluated. The optical system is capable of monitoring absorbance, fluorescence and light scattering reactions. In addition, an ion selective electrode unit may be incorporated for the measurement of sodium, potassium and chloride.

The precision, accuracy, linearity, calibration stability and carry-over were investigated for 19 routine chemistries. The within-batch and between-day precision data were good in the majority of cases; some chemistries demonstrated poor performance at low analyte concentrations. Method comparison studies showed good agreement, with small discrepancies being due to different calibration material and methodological differences. Major discrepancies were found with CK and LD; linearity studies were good in all cases, except calcium. No significant sample or reagent carry-over was found.

Assessment of throughput for a variety of test profiles varied between 300 and 605 tests per hour.

The instrument was easy to operate, very flexible and capable of handling a large and varied workload.


Journal of Automatic Chemistry Vol. 11, No. 1 (January–February 1989), pp. 15–21

### An evaluation of the Monarch chemistry analyser

**D. J. Berry and C. P. Price**

*Department of Clinical Biochemistry, Addenbrooke's Hospital, Hills Road, Cambridge CB2 2QR, UK*

*The Monarch Chemistry system, a centrifugal analyser incorporating sophisticated robotics for analytical rotor transfer and flexible software for workload scheduling, has been evaluated. The optical system is capable of monitoring absorbance, fluorescence and light scattering reactions. In addition, an ion selective electrode unit may be incorporated for the measurement of sodium, potassium and chloride.*

*The precision, accuracy, linearity, calibration stability and carry-over were investigated for 19 routine chemistries. The within-batch and between-day precision data were good in the majority of cases; some chemistries demonstrated poor performance at low analyte concentrations. Method comparison studies showed good agreement, with small discrepancies being due to different calibration material and methodological differences. Major discrepancies were found with CK and LD; linearity studies were good in all cases, except calcium. No significant sample or reagent carry-over was found.*

*Assessment of throughput for a variety of test profiles varied between 300 and 605 tests per hour.*

*The instrument was easy to operate, very flexible and capable of handling a large and varied workload.*

### Introduction

The centrifugal analyser was first described by Dr Norman Anderson in 1969 [1]. The first commercial instrument was released in 1978 and since then several instruments have been developed based on this technology [2]. The recent development of the Monarch chemistry analyser features a number of new innovations in line with predictions made by Tiffany [3]. To virtually eliminate operator intervention, the analyser incorporates a transport arm to transfer analytical rotors from the feed-stack through loading, analysis and finally to the discard stack. The optical system is capable of changing optical filters in less than 5 s, enabling more than one chemistry to be run within the same rotor. In addition, the system provides a sophisticated software package which includes an intelligent work scheduling system.

The analytical performance of the Monarch was assessed for 19 different analytes. The aspects evaluated included within-day and between-day precision at three different analyte concentrations; calibration stability; method linearity; relative accuracy and system carry-over [4].

### Materials and methods

### The instrument

The Monarch is a single free-standing unit requiring a 13 amp power supply; diluent and waste bottles are self-contained within the system. The instrument is capable of performing up to a maximum of 24 tests per sample. An optional ISE unit allows the measurement of sodium, potassium and chloride, which are always made together. The sample throughput for a variety of chemistry profiles varies between 300 and 605 tests per hour. Stat requests can be processed at any time during a routine run; when completed the instrument returns to the original request. The optical system allows the measurement by absorbance, fluorescence and light-scattering techniques. Either rate or end-point assays, with up to four reagent additions, can be monitored.

Samples and reagents are housed in a compartment maintained at a temperature of 15 °C for greater reagent stability. Reagent boats are identified by a bar-coded label which is read by an optical bar-code sensor.

Sample and reagent are loaded into a disposable UVT rotor via two stainless-steel pipette tips attached to the pipette arm. Each rotor contains 39 cuvettes into which sample (89 µl maximum) and reagent (100–236 µl) are dispensed. The pipette arm is located in a thermal box, allowing movement between the reagent compartment and analysis compartment. The instrument uses two diluent-filled syringes to load the rotor. During loading the syringes are drawn down, resulting in sample and reagent being taken up into the tubing connected to the pipette tips. After sampling, the pipette arm moves back to the home position and sample and reagent are dispensed into the rotor. The pipette arm is heated to the same temperature as the analysis compartment (25, 30 or 37 °C).

The analysis compartment contains a robotic transport arm surrounded by a feed-stack, a loading table, analysis table and discard/park table. The feed-stack contains a supply of clean rotors. The optics module contains the tungsten and xenon lamps, a scanning monochromator and sets of mirrors, lenses and optical windows. Wavelengths between 340 and 690 nm can be selected.

The transport arm transfers the loaded rotor to the analysis table, where reagents and sample are mixed and data acquisition takes place. When the analysis is complete a full rotor is discarded and a partially used rotor is held on the park station, if possible, for re-use.

0142–0453/89 $3.00 © 1989 Taylor & Francis Ltd.

The ISE module is housed independently within the system. Ion-selective electrodes determine the concentration of sodium, potassium and chloride in plasma, serum, urine or sweat. The sample is diluted, mixed and then drawn into the electrode module, which contains the sodium, potassium, chloride and reference assemblies.

The analyser is controlled by a computer with which the operator communicates via a keyboard and VDU. Two disks store test parameters, response data, results, user file data, utilities and diagnostic information.

The software contains a number of features which allow the user to review, edit and print information. Each test has a corresponding parameter table which may be readily accessed by the user. New tests may also be created in this way.

The workload may be scheduled in one of two ways – either time-optimized or patient priority. In time-optimized mode the instrument schedules the request for optimum throughput by analysing batches of chemistries together. This is the most time-efficient and economical mode of operation. In patient priority mode, the system will schedule to analyse the tests sample by sample.

To increase the efficiency of the instrument and allow more than one chemistry to be run on one rotor, tests are assigned to a compatibility class. For tests to be in the same compatibility class the main characteristics of the class must be the same.

*Analytical methods and reagents*

Details of the methods employed in the evaluation are shown in table 1. All reagents were prepared and used according to the manufacturers' recommendations. The calibrators used were RefIL A, B, C (Instrumentation Laboratory) and for the calibration of BCP albumin, Nycomed Reference Material. A range of quality-control sera were used: Serachem Level I and II (Fisher Diagnostics), Technicon Reference (Technicon Instruments), Autonorm Low and High (Nycomed) and Precinorm E and Precipath E (Boehringer Mannheim)

*Precision*

The within-batch precision was assessed by analysing control sera at three different analyte concentrations. Eighteen samples of each control sera were assayed, thus ensuring that all samples would be analysed within one rotor. This was carried out using the instrument in both 'time-optimized' and 'patient-priority' mode.

The between-batch precision was assessed over a period of 20 working days. Quality-control material was reconstituted at the beginning of each day.

*Calibration stability*

The instrument was calibrated at the start of each day, according to the manufacturers' recommendations. Calibrators and control sera were then assayed immediately following calibration and at the end of each day.

*Linearity*

Samples known to contain high levels of analyte were diluted in varying proportions with 60 g/l BSA. Where this was not possible a commercial lyophilized quality control material was reconstituted in a smaller volume than recommended to give elevated analyte concentrations. For the electrolytes, a stock solution of sodium

*Table 1. Details of methods employed on Monarch and comparison system.*

| *Analyte* | *Sample volume (μl)* | *Principle of Monarch method* | *Comparison method* |
|---|---|---|---|
| Sodium, Potassium, Chloride | 30 | ISE | Flame photometry (Na, K only) SMAII |
| $TCO_2$ | 3 | Enzymatic, phosphoenolpyruvate carboxylase | Indicator dye, SMAII |
| Glucose | 3 | Hexokinase, glucose-6-phosphate dehydrogenase | Glucose oxidase SMAII |
| Urea | 3 | Urease/GLDH | Diacetyl monoxime SMAII |
| Creatinine | 9 | Picric acid | Jaffe SMAII |
| Total protein | 5 | Biuret | Biuret SMAII |
| Albumin | 3 | Bromocresol purple | Bromocresol purple SMAII |
| Calcium | 5 | Cresolphthalein complexone | Cresolphthalein complexone SMAII |
| Phosphate | 4 | Ammonium molybdate | Phosphomolybdate, SMAII |
| Bilirubin | 8 | Sulphanilic acid | Jendrassik and Grof, SMAII |
| ALP | 10 | p-nitrophenyl phosphate with DEA buffer | p-nitrophenyl phosphate with AMP buffer, SMAII |
| ALT | 20 | L-alanine, α-ketoglutarate | L-alanine optimized SMAII |
| CK | 10 | Creatine phosphate | Creatine phosphate, Multistat III |
| LD | 5 | Pyruvate → lactate | Pyruvate → lactate, Multistat III |
| Urate | 20 | Uricase, 340 nm | Uricase 340 nm, RA1000 |
| Cholesterol | 3 | Cholesterol oxidase | Cholesterol oxidase, RA1000 |
| Triglyceride | 3 | Lipase, glycerophosphate oxidase | Lipase, glycerophosphate oxidase |

chloride was used for sodium and chloride and a solution of potassium chloride for potassium. Further dilutions of the stock were made in deionized water.

*Accuracy*

At least 100 patient samples were analysed on the Monarch and the results compared to those obtained in the routine laboratory. Details of comparison methods are given in table 1.

*Carry-over*

The design of the instrument required investigation of both 'sample to sample' carry-over and 'reagent to sample or reagent' carry-over.

*Sample to sample carry-over*

Three sequences of a high pool followed by a low pool were assayed for each analyte. The mean carry-over was calculated for each sequence using the formula:

$$\frac{L_1 - L_3}{H_3 - L_3} \times 100\,(\%)$$

*Reagent to sample or reagent carry-over*

A mid-level human serum pool was assayed, in triplicate, such that, ultimately each chemistry had been preceded and followed by the other. The coefficient of variation for the analyte being investigated was then calculated.

### Results and discussion

*Precision*

Results for the within-batch and between-day precision are shown in tables 2 and 3 respectively.

The results for the within-batch precision shows good performance in the majority of cases. The disappointing precision obtained at low levels of urea, ALT and triglyceride may be due to the low absorbance changes for these assays. Generally, the precision obtained whilst scheduling the instrument in 'patient-priority' mode was inferior to that found in time-optimized mode. This may be due to each test having its own blank in patient-priority mode, as opposed to one blank for a batch of tests in time-optimized mode.

The day-to-day precision was found to be acceptable, with the exception of creatinine, calcium, CK and triglyceride, which were disappointing. The performance of the $TCO_2$ method was disappointing throughout the concentration range.

There was a definite improvement in the precision obtained for CK and LD when using alternative quality-control material.

The performance of the ISE unit was very good.

*Table 2. Within-batch precision at three analyte concentrations. Results for time-optimized and patient-prioritized mode (in parentheses).*

| *Analyte* | *Mean* | *SD* | *CV(%)* | *CV(%)* |
|---|---|---|---|---|
| Sodium (mmol/l) | 123·6 | 0·88 | 0·72 | (0·34) |
| | 139·1 | 0·66 | 0·48 | (0·49) |
| | 147·9 | 0·59 | 0·40 | (0·37) |
| Potassium (mmol/l) | 1·90 | 0·00 | 0·00 | — |
| | 4·30 | 0·00 | 0·00 | (1·18) |
| | 6·53 | 0·05 | 0·69 | (0·76) |
| Chloride (mmol/l) | 91·0 | 0·56 | 0·62 | (0·29) |
| | 98·7 | 0·38 | 0·38 | (0·46) |
| | 116·5 | 0·36 | 0·31 | (0·37) |
| $TCO_2$ (mmol/l) | 15·8 | 0·39 | 2·47 | (3·72) |
| | 25·4 | 0·39 | 1·55 | (4·71) |
| | 27·2 | 0·67 | 2·47 | (4·57) |
| Glucose (mmol/l) | 4·09 | 0·04 | 1·07 | (1·96) |
| | 12·30 | 0·14 | 1·12 | (2·17) |
| | 19·79 | 0·20 | 1·01 | (1·86) |
| Urea (mmol/l) | 5·52 | 0·20 | 3·55 | (3·94) |
| | 18·22 | 0·26 | 1·42 | (2·79) |
| | 19·11 | 0·35 | 1·19 | (3·68) |
| Creatinine (μmol/l) | 101·9 | 2·06 | 2·02 | (3·01) |
| | 452·6 | 4·10 | 0·91 | (2·21) |
| | 713·8 | 5·25 | 0·74 | (2·40) |
| Total protein (g/l) | 40·9 | 0·48 | 1·17 | (2·58) |
| | 67·6 | 0·78 | 1·15 | (2·05) |
| Albumin (g/l) | 24·3 | 0·34 | 1·42 | — |
| | 38·0 | 0·23 | 0·60 | (1·68) |
| | 49·2 | 0·25 | 0·51 | (1·29) |
| Calcium (mmol/l) | 1·02 | 0·02 | 1·63 | — |
| | 2·42 | 0·03 | 1·63 | — |
| | 3·19 | 0·02 | 0·66 | (1·38) |
| Phosphate (mmol/l) | 1·17 | 0·02 | 1·36 | (3·16) |
| | 1·67 | 0·02 | 1·04 | (2·49) |
| | 2·84 | 0·02 | 0·70 | (2·48) |
| T Bilirubin (μmol/l) | 11·9 | 0·24 | 2·01 | (2·23) |
| | 64·9 | 2·61 | 4·03 | (3·20) |
| | 147·7 | 0·61 | 0·41 | (1·38) |
| ALP (I.U./l) | 74·3 | 0·98 | 1·32 | (3·49) |
| | 170·8 | 1·39 | 0·81 | (2·11) |
| | 644·7 | 6·00 | 0·93 | (1·66) |
| ALT (I.U./l) | 17·7 | 0·72 | 4·06 | (6·12) |
| | 74·7 | 0·81 | 1·09 | (1·67) |
| | 189·0 | 1·10 | 0·58 | (1·86) |
| CK (I.U./l) | 121·2 | 1·59 | 1·31 | (1·88) |
| | 245·6 | 3·08 | 1·25 | — |
| | 411·4 | 6·56 | 1·59 | (7·88) |
| LD (I.U./l) | 228·9 | 4·85 | 2·12 | (3·49) |
| | 370·4 | 5·76 | 1·55 | — |
| | 622·4 | 5·54 | 0·89 | (1·76) |
| Urate (μmol/l) | 200·8 | 2·16 | 1·07 | (1·76) |
| | 327·0 | 5·98 | 1·48 | — |
| | 588·0 | 2·29 | 0·39 | (1·10) |
| Cholesterol (mmol/l) | 2·56 | 0·03 | 1·23 | (2·54) |
| | 4·83 | 0·06 | 1·32 | (2·88) |
| | 5·82 | 0·07 | 1·22 | — |
| Triglyceride (mmol/l) | 0·26 | 0·015 | 5·71 | (14·2) |
| | 0·78 | 0·040 | 5·10 | — |
| | 2·23 | 0·040 | 1·82 | — |

*Calibration stability*

Assaying quality control material at the beginning and end of each day showed deterioration in the performance of some analytes. In these cases more frequent calibration may be required.

Reviewing the absorbance data obtained for each of the calibrators over the period of the evaluation showed significant variation in absorbance for $TCO_2$, urea and calcium.

*Table 3. Between-batch precision at three analyte concentrations for controls analysed immediately following calibration. Results for controls analysed in the afternoon are given in parentheses.*

| *Analyte* | *Mean* | *SD* | *CV (%)* | *CV (%)* |
|---|---|---|---|---|
| Sodium (mmol/l) | 123·4 | 1·14 | 0·92 | (1·00) |
| | 140·6 | 1·65 | 1·17 | (0·88) |
| | 150·0 | 1·45 | 0·97 | (1·05) |
| Potassium (mmol/l) | 1·90 | 0·00 | 0·00 | (0·00) |
| | 4·30 | 0·04 | 0·88 | (1·40) |
| | 6·59 | 0·07 | 1·02 | (1·29) |
| Chloride (mmol/l) | 88·0 | 0·93 | 1·05 | (1·31) |
| | 98·9 | 1·13 | 1·14 | (1·67) |
| | 112·1 | 1·19 | 1·06 | (1·90) |
| $TCO_2$ (mmol/l) | 18·9 | 1·29 | 6·90 | (10·40) |
| | 28·0 | 1·65 | 5·91 | (8·10) |
| | 32·8 | 2·07 | 6·32 | (9·09) |
| Glucose (mmol/l) | 4·12 | 0·12 | 2·56 | (2·70) |
| | 12·41 | 0·38 | 3·09 | (2·44) |
| | 19·91 | 0·43 | 2·16 | (2·62) |
| Urea (mmol/l) | 6·03 | 0·19 | 3·10 | (4·02) |
| | 19·90 | 0·58 | 2·91 | (2·88) |
| | 29·59 | 1·14 | 3·84 | (5·15) |
| Creatinine (μmol/l) | 106·3 | 8·05 | 7·60 | (4·70) |
| | 452·8 | 8·48 | 1·87 | (2·38) |
| | 718·1 | 11·91 | 1·66 | (2·59) |
| Total protein (g/l) | 39·5 | 0·75 | 1·90 | (2·88) |
| | 67·1 | 1·21 | 1·81 | (2·52) |
| | 82·4 | 1·53 | 1·85 | (1·77) |
| Albumin (g/l) | 24·2 | 0·44 | 1·81 | (2·63) |
| | 38·8 | 1·10 | 2·86 | (3·19) |
| | 49·0 | 0·46 | 0·94 | (1·80) |
| Calcium (mmol/l) | 1·03 | 0·058 | 5·59 | (3·48) |
| | 2·48 | 0·042 | 1·70 | (3·07) |
| | 3·25 | 0·050 | 1·53 | (2·93) |
| Phosphate (mmol/l) | 1·11 | 0·022 | 1·95 | (3·13) |
| | 1·64 | 0·036 | 2·17 | (3·21) |
| | 2·72 | 0·054 | 1·99 | (3·37) |
| T Bilirubin (μmol/l) | 10·8 | 0·83 | 7·70 | (6·70) |
| | 60·0 | 1·50 | 2·50 | (4·00) |
| | 149·9 | 3·72 | 2·48 | (2·64) |
| ALP (I.U./l) | 80·3 | 2·63 | 3·27 | (3·37) |
| | 176·8 | 5·16 | 2·92 | (2·24) |
| | 653·9 | 15·70 | 2·40 | (2·21) |
| ALT (I.U./l) | 22·7 | 1·49 | 6·60 | (6·40) |
| | 80·3 | 1·92 | 2·39 | (3·27) |
| | 192·3 | 3·09 | 1·61 | (1·58) |
| CK (I.U./l) | 119·6 | 10·15 | 8·50 | (8·12) |
| | 226·1 | 21·30 | 9·41 | (11·00) |
| | 445·8 | 37·04 | 8·31 | (5·48) |
| LD (I.U./l) | 236·7 | 7·00 | 2·97 | (3·20) |
| | 392·0 | 16·80 | 4·28 | (3·57) |
| | 631·5 | 20·73 | 3·28 | (3·11) |
| Urate (μmol/l) | 210·0 | 5·22 | 2·48 | (4·37) |
| | 334·3 | 6·87 | 2·05 | (3·96) |
| | 583·0 | 11·37 | 1·95 | (3·76) |
| Cholesterol (mmol/l) | 2·65 | 0·079 | 3·00 | (2·85) |
| | 4·87 | 0·116 | 2·40 | (2·40) |
| | 6·03 | 0·150 | 2·47 | (4·29) |
| Triglyceride (mmol/l) | 0·62 | 0·050 | 8·13 | (4·39) |
| | 2·11 | 0·097 | 4·62 | (2·99) |
| | 5·56 | 0·190 | 3·38 | (2·78) |

Assessing calibration stability by calculation of the analyte concentration based on day 1 calibration figures showed a variation of greater than 4SD in the case of calcium, phosphate, total protein and urate.

*Table 4. Linearity of assays performed on the Monarch.*

| *Analyte* | *Determined range of linearity* |
|---|---|
| Sodium | 110–160 mmol/l |
| Potassium | 1–10 mmol/l |
| Chloride | 70–140 mmol/l |
| $TCO_2$ | 0–45 mmol/l |
| Glucose | 0–30 mmol/l |
| Urea | 0–35 mmol/l |
| Creatinine | 0–1500 μmol/l |
| Total protein | 0–130 g/l |
| Albumin | 0–50 g/l |
| Calcium | 0–2·5 mmol/l |
| Phosphate | 0–4·5 mmol/l |
| Bilirubin | 0–500 μmol/l |
| ALP | 0–1500 IU/l |
| ALT | 0–450 IU/l |
| CK | 0–1000 IU/l |
| LD | 0–750 IU/l |
| Urate | 0–0·9 mmol/l |
| Cholesterol | 0–10·5 mmol/l |
| Triglyceride | 0–10·5 mmol/l |

### *Linearity*

The linear range for each analyte is shown in table 4. The results obtained agreed with the expected ranges for each analyte, except in the case of calcium. The results suggest that the assay is not linear above 3·0 mmol/l.

### *Accuracy*

The method comparison studies indicated a good agreement in the majority of cases (table 5 and figure 1). Small differences were attributable to the use of different calibration materials. Major discrepancies were found with amylase and alkaline phosphatase, due to the use of different methods, and with CK and LD. Experiments were carried out in an attempt to determine the cause of

*Table 5. Linear regression statistics for Monarch (y-axis) against various comparison methods (x-axis).*

| *Test* | *N* | *Slope* | *Y-intercept* | *Correlation coefficient* |
|---|---|---|---|---|
| Sodium | 176 | 1·126 | −16·14 | 0·987 |
| Potassium | 148 | 0·979 | 0·357 | 0·996 |
| $TCO_2$ | 121 | 0·925 | −4·37 | 0·837 |
| Glucose | 161 | 0·975 | −0·049 | 0·997 |
| Urea | 188 | 1·012 | 0·60 | 0·996 |
| Creatinine | 170 | 0·926 | 25·57 | 0·997 |
| Total protein | 137 | 0·928 | 4·33 | 0·974 |
| Albumin | 105 | 0·933 | 3·057 | 0·984 |
| Calcium | 139 | 0·981 | 0·035 | 0·962 |
| Phosphate | 123 | 1·025 | −0·068 | 0·980 |
| Total bilirubin | 161 | 0·975 | −0·479 | 0·997 |
| ALP | 156 | 1·571 | −0·874 | 0·989 |
| ALT | 145 | 0·925 | −0·057 | 0·994 |
| CK | 161 | 0·827 | 0·568 | 0·999 |
| LD | 160 | 0·725 | 17·27 | 0·996 |
| Urate | 118 | 0·935 | −0·002 | 0·985 |
| Cholesterol | 141 | 1·095 | 0·069 | 0·990 |
| Triglyceride | 114 | 0·991 | −0·04 | 0·995 |

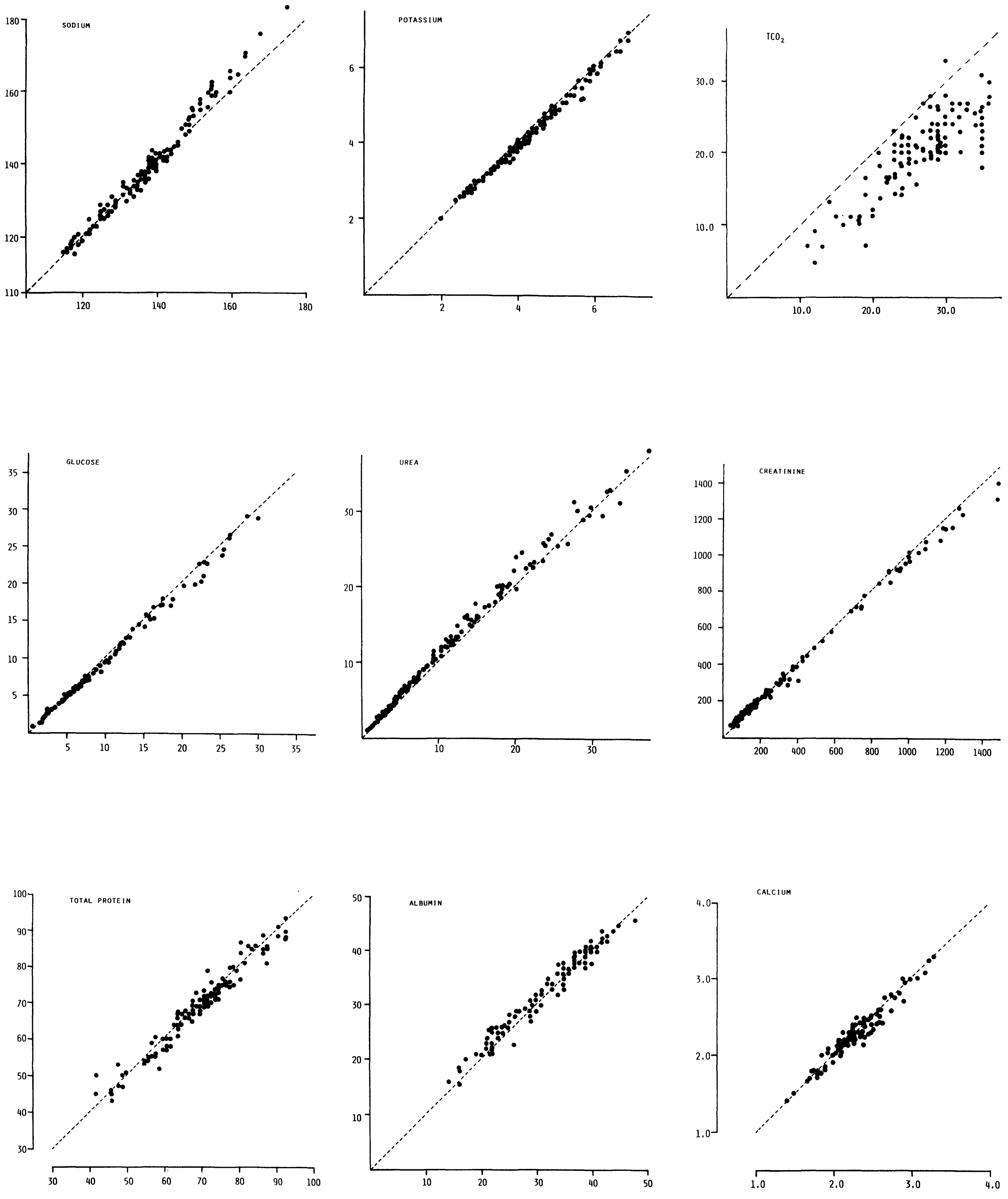

*Figure 1. Comparison of results for analytes measured on the Monarch (y-axis) and comparison method (x-axis). Details of comparison methods are given in table 1.*

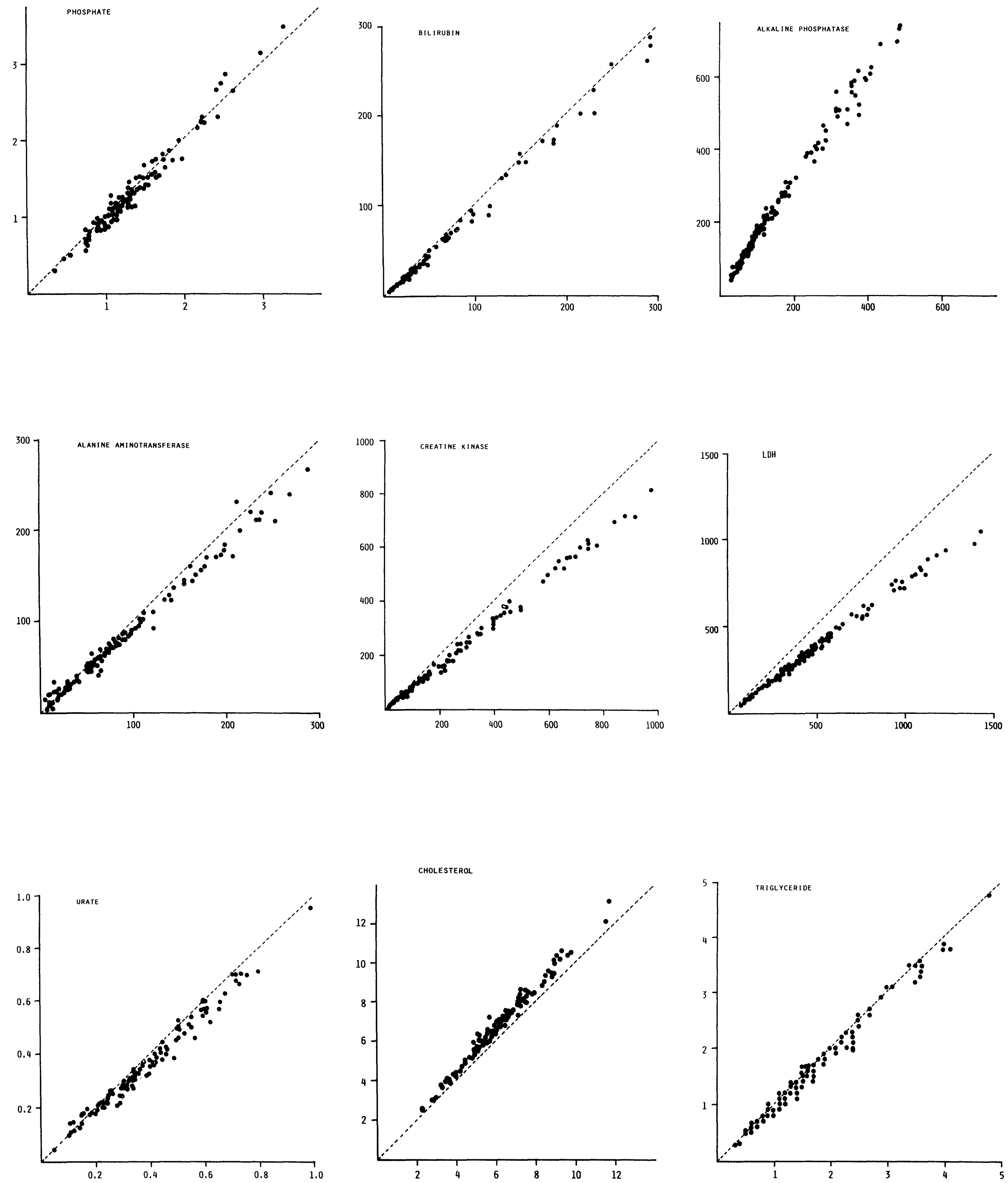

*Figure 1 continued.*

*Table 6. Experiment to determine recovery of aqueous and serum based sample pipetting on the Monarch.*

| *Sample* | **Measured absorbance* | *Expected absorbance* | *Recovery (%)* |
|---|---|---|---|
| *NADH:* (μl) | | | |
| 3 | 0·1863 | 0·1922 | 97 |
| 6 | 0·3602 | 0·3817 | 94 |
| 9 | 0·5367 | 0·5739 | 94 |
| 12 | 0·7184 | 0·7567 | 95 |
| *Glucose* in 60% Albumin: Concentration (mmol/l) | | | |
| 5 | 0·344 | 0·403 | 85 |
| 10 | 0·664 | 0·806 | 84 |
| 15 | 1·069 | 1·209 | 88 |

* NADH: mean value of five determinations. Glucose: mean value of three determinations.

*Table 7. Sample to sample carry-over on the Monarch.*

| *Analyte* | *Mean high level (H3)* | *Mean low level (L3)* | *Mean L1–L3* | *Mean carry-over (%)* |
|---|---|---|---|---|
| Sodium (mmol/l) | 258·1 | 99·5 | 0·1 | 0·06 |
| Potassium (mmol/l) | 12·8 | 1·9 | 0 | 0 |
| Chloride (mmol/l) | 180·8 | 91·8 | 1·7 | 1·9 |
| $TCO_2$ (mmol/l) | 57·6 | 3·8 | 0·4 | 0·70 |
| Glucose (mmol/l) | 35·5 | 2·1 | 0·1 | 0·30 |
| Urea (mmol/l) | 44·8 | 2·6 | 0·2 | 0·47 |
| Creatinine (μmol/l) | 1246 | 58 | 3 | 0·25 |
| Total protein (g/l) | 136·8 | 30·3 | 0·6 | 0·56 |
| Albumin (g/l) | 42·2 | 15·5 | 0·3 | 1·12 |
| Calcium (mmol/l) | 4·29 | 1·20 | 0·01 | 0·32 |
| Phosphate (mmol/l) | 5·30 | 0·34 | 0·01 | 0·20 |
| Total bilirubin (μmol/l) | 823 | 4·8 | 0·40 | 0·05 |
| ALP (IU/l) | 1200 | 52 | 1·00 | 0·09 |
| ALT (IU/l) | 2312 | 18 | 5·0 | 0·22 |
| CK (IU/l) | 4000 | 43 | 2·0 | 0·05 |
| LD (IU/l) | 1360 | 103 | 2·0 | 0·16 |
| Urate (μmol/l) | 1·05 | 0·152 | 0·005 | 0·56 |
| Cholesterol (mmol/l) | 11·08 | 1·27 | 0·06 | 0·61 |
| Triglyceride (mmol/l) | 10·0 | 0·74 | 0·07 | 0·76 |

the discrepancy found with CK and LD assays. Solutions of NADH were prepared and loaded by the instrument using 3, 6, 9 and 12 μl sample volumes. The experiment was also carried out using 5, 10 and 15 mmol/l glucose in 60% albumin using an end-point glucose dehydrogenase method adapted to the Monarch. The expected absorbances (determined from the extinction coefficient of NADH) at 340 nm were confirmed with similar experiments using externally, manually loaded rotors (table 6).

*System carry-over*

*Sample carry-over* – the mean carry-over obtained in all cases was negligible (table 7).

*Reagent carry-over* – the coefficient of variation obtained was less than 4SD (mean value obtained in the within-batch precision study) in all cases except two. The experiment was therefore repeated using three sequences of the initial procedures, for CK into creatinine and triglyceride into LD. No significant carry-over was detected.

### Conclusion

The Monarch chemistry analyser demonstrated good performance over a wide range of analytes. The coefficients of variation obtained for the within-batch and between-day precision data were good, although the performance of the $TCO_2$ and calcium methods were disappointing.

The linearity of the methods were sufficiently broad to allow measurements over a wide range without the necessity to dilute samples. There was no significant carry-over.

For the majority of the methods calibration would only be required once a day, and, in many cases, weekly calibration would be acceptable.

Comparison between the Monarch methods and those used in the routine laboratory were good in the majority of cases. The discrepancy seen in the enzyme results could not be resolved and it is currently necessary to employ a factor to correct for the difference.

The Monarch is capable of handling a reasonably large and varied workload. The work organization of the instrument is most efficient when batches of tests are analysed together. In this way a discretionary approach to testing can be achieved without affecting the performance of the instrument. Stat samples can be given priority at any time during a routine run.

We found the instrument to be flexible and easy to use, requiring a minimum of training. Only simple maintenance procedures were required on a daily, weekly and monthly basis.

### References

1. Anderson, N. G., *American Journal of Clinical Pathology*, **53** (1970), 778.
2. Price, C. P. and Spencer, K. (Eds) In *Centrifugal Analysers in Clinical Chemistry*, edited by Price, C. P. and Spencer, K. (Praeger, Eastbourne, 1980), p. 5.
3. Tiffany, T. O. In *Centrifugal Analysers in Clinical Chemistry*, edited by Price, C. P. and Spencer, K. (Praeger, Eastbourne, 1980), p. 3.
4. Broughton, P. M. G., Gowenlock, A. H., McCormack, J. J. and Neill, D. W., *Annals of Clinical Biochemistry*, **11** (1974), 207.

